# Comparing the Genetic Diversity and Antimicrobial Resistance Profiles of *Campylobacter jejuni* Recovered from Cattle and Humans

**DOI:** 10.3389/fmicb.2017.00818

**Published:** 2017-05-09

**Authors:** Wonhee Cha, Rebekah E. Mosci, Samantha L. Wengert, Cristina Venegas Vargas, Steven R. Rust, Paul C. Bartlett, Daniel L. Grooms, Shannon D. Manning

**Affiliations:** ^1^Department of Microbiology and Molecular Genetics, Michigan State University, East LansingMI, USA; ^2^Department of Large Animal Clinical Sciences, Michigan State University, East LansingMI, USA; ^3^Department of Animal Science, Michigan State University, East LansingMI, USA

**Keywords:** *Campylobacter jejuni*, antimicrobial resistance, cattle, multilocus sequence typing, molecular epidemiology of infectious diseases, zoonotic pathogen

## Abstract

*Campylobacter jejuni*, a leading cause of gastroenteritis in humans, is a foodborne pathogen that can reside in chickens, pigs, and cattle. Because resistance to fluoroquinolones and macrolides, which are commonly used to treat human infections, has emerged in *C. jejuni*, it is imperative to continously monitor resistance patterns and examine the genetic variation in strains from human infections and animal reservoirs. Our previous study of *C. jejuni* from human campylobacteriosis cases showed a significantly higher rate of tetracycline resistance compared to national trends, and identified multilocus sequence type (ST)-982 and a history of cattle contact to be associated with tetracycline resistance. To further investigate these associations, we conducted a cross-sectional study to determine the frequency of antimicrobial resistance and examine the genetic diversity of *C. jejuni* recovered from 214 cattle at three Michigan herds. Overall, the prevalence of *C. jejuni* was 69.2% (range: 58.6–83.8%) for the three farms, and 83.7% (*n* = 113) of isolates were resistant to one or more antimicrobials. Resistance to only tetracycline predominated among the cattle isolates (*n* = 89; 65.9%) with most resistant strains belonging to ST-459 (96.5%) or ST-982 (86.4%). Among the 22 STs identified, STs 459 and 982 were more prevalent in one feedlot, which reported the use of chlortetracycline in feed upon arrival of a new herd. PCR-based fingerprinting demonstrated that the ST-982 isolates from cattle and humans had identical banding patterns, suggesting the possibility of interspecies transmission. Resistance to macrolides (1.5%) and ciprofloxacin (16.3%) was also observed; 14 of the 22 ciprofloxacin resistant isolates represented ST-1244. Together, these findings demonstrate a high prevalence of antimicrobial resistant *C. jejuni* in cattle and identify associations with specific genotypes. Continuous monitoring and identification of risk factors for resistance emergence are imperative to develop novel methods aimed at decreasing pathogen persistence in food animal reservoirs and the frequency of resistant infections in humans.

## Introduction

*Campylobacter jejuni* is one of the most common causes of human gastroenteritis in the U.S., affecting roughly 1.3 million people annually (Scallan et al., 2011). Most *Campylobacter* isolates recovered from patients with gastroenteritis are classified as *C. jejuni* (89%) followed by *C. coli* (8%) (Centers for Disease Control and Prevention [CDC], 2014a). *Campylobacter* spp. can asymptomatically colonize the intestines of various food animals including chickens, cattle, and pigs (Altekruse et al., 1999; Harvey et al., 1999; Stanley and Jones, 2003). Previous studies have been more focused on chickens, as they are considered to be the major reservoir for human campylobacteriosis cases (Harris et al., 1986; Fitch et al., 2005). Recent molecular genotyping and statistical modeling studies (Pritchard et al., 2000; Wilson et al., 2008; Sheppard et al., 2009), however, have shown that cattle also represent an important source for human infections. In Finland and the U.K., for instance, cattle were found to contribute equally to human infections when compared to chickens (Wilson et al., 2008; de Haan et al., 2010). Another study conducted by the Centers for Disease Prevention and Control (CDC) reported that dairy products, mostly raw milk and cheese, contributed to 66% of the campylobacteriosis outbreaks in the U.S. (Interagency Food Safety Analytics Collaboration Project, 2015). *Campylobacter* shed by cattle can also contribute to contamination of the environment via run-off water from farming and meat processing operations, which represent additional sources for human infections. One prior study found identical genotypes of *C. jejuni* recovered from dairy cattle and ground water (Kwan et al., 2008), while our study of 7,182 human campylobacteriosis cases in Michigan identified epidemiological associations between disease and contact with ruminants, well water, and living in a rural area with higher cattle densities (Cha et al., 2016a).

A major concern with regard to treating campylobacteriosis in humans is antimicrobial resistance, particularly resistance to the fluoroquinolones and macrolides, which has increased significantly over the past two decades (Centers for Disease Control and Prevention [CDC], 2014b). Because of the association between fluoroquinolone use in poultry and increasing rates of resistance in human infections, fluroquinolones were banned for use in poultry in the U.S. in 2005 (Nelson et al., 2007). Nonetheless, increasing frequencies of fluoroquinolone resistant *Campylobacter* have been reported in both chickens and humans (Centers for Disease Control and Prevention [CDC], 2014b; United States Department of Agriculture [USDA], 2014), though few studies have been conducted in cattle. Fluoroquinolones such as enrofloxacin, were licensed for use in beef cattle in 1998 (Anderson et al., 2001) and macrolides including tulathromycin and tilmicosin, are commonly used for treating respiratory diseases in both beef and dairy cattle (Cahn and Line, 2010; United States Department of Agriculture [USDA], 2013).

The use of tetracyclines, like chlortetracycline or oxytetracycline, is also common in the cattle industry. According to the national study conducted by the U.S. Department of Agriculture (USDA) in 2007, over one-half (57.5%) of dairy operations in the U.S. were feeding medicated milk replacer, often containing tetracycline, to calves and pre-weaned heifers (Animal and Plant Health Inspection Service [APHIS], 2008). Tetracycline was also reported to be the primary antimicrobial used for treating lameness. It is noteworthy that our prior study of *C. jejuni* strains from Michigan patients demonstrated a significantly higher rate of tetracycline resistance (61.7%) compared to the national average (47.8%) (χ^2^ *P* < 0.01) and resistance frequencies were higher in cases reporting contact with ruminants, specifically cattle, prior to the onset of symptoms (Cha et al., 2016b). We also demonstrated that strains belonging to multilocus sequence type (ST)-982 were more likely to be resistant to tetracycline and associated with infections among cases reporting ruminant contact. Together, these data suggest that a pathogenic, tetracycline-resistant lineage may be circulating in the Michigan cattle population. To investigate this possibility, we sought to examine the genetic diversity and antimicrobial resistance profiles of cattle-derived *C. jejuni*, which were collected during the same time period as the strains associated with human infections. We hypothesized that there was a high prevalence of *C. jejuni* in cattle and that the strains had a similar frequency of tetracycline resistance and resistance to other important antimicrobials when compared to the human strains. We also hypothesized that similar genotypes with identical antimicrobial resistance profiles could be detected in both cattle and humans.

## Materials and Methods

### Sample Collection and *C. jejuni* Identification

Fecal samples were collected from 214 cows at one dairy (Farm A) and two beef herds (Farms B and C) in mid-Michigan in 2012 as part of an epidemiologic study to investigate fecal shedding of Shiga toxin-producing *Escherichia coli* in cattle (Venegas Vargas et al., 2016). A questionnaire, which was administered by interviewing each farm manager, was used to obtain data regarding farm demographics, management practices, and herd health management strategies (Supplementary Table S1). The distance from Farm A to Farm C was about 62 miles, while Farm B was located roughly in between the two farms. The average age of cows from Farm A was 3.9 years while all beef cows were ∼1 year old. This study was carried out in accordance with the recommendations and approval by the Institutional Animal Care and Use Committee of Michigan State University (AN12/10-223-00).

To isolate *Campylobacter* from cattle, 10 μl of each fecal sample was directly plated on blood agar containing cefoperazone (20 μg/ml), vancomycin (20 μg/ml), and amphotericin B (4 μg/ml) for 48 h at 37°C in microaerophilic conditions using the Oxoid^TM^ CampyGen (Thermo Scientific, Waltham, MA, USA). Single colonies were selected from each sample based on morphology and appearance while focusing on small pinpoint gray colonies without hemolysis. After growth at 37°C for 48 h using the same conditions, DNA was extracted using the Wizard^®^ genomic DNA purification kit (Promega, Madison, WI, USA) and stored at -20°C. Confirmation and classification of *Campylobacter* were performed by multiplex PCR as described (Yamazaki-Matsune et al., 2007); all strains were stored in trypticase soy broth with 10% glycerol at -80°C until use. A previously characterized set of 94 *C. jejuni* clinical strains from humans with campylobacteriosis were used for comparison (Cha et al., 2016b). These human strains, which were recovered between 2011 and 2012 by the Michigan Department of Health and Human Services, were compared to the cattle-derived strains in this study. All of the human strains were cultured on the same agar media as the cattle samples and were confirmed with multiplex PCR.

### Antimicrobial Susceptibility Profiling and Determination of *tet(O)* Gene

As described previously (Cha et al., 2016b), the minimum inhibitory concentrations (MICs) of nine antimicrobials were determined using broth microdilution with the CAMPY plate from Sensititre (Trek Diagnostic Systems, Cleveland, OH, USA) according to the manufacturer’s instruction. Antimicrobials included ciprofloxacin, nalidixic acid, azithromycin, erythromycin, tetracycline, florfenicol, telithromycin, clindamycin, and gentamicin. *C. jejuni* ATCC 33560 was used as a control and the antimicrobial breakpoints were determined using the epidemiologic cut-off values (ECOFFs) following the guidelines of the European Committee on Antimicrobial Susceptibility Testing [EUCAST] (2015) and National Antimicrobial Resistance Monitoring System (Centers for Disease Control and Prevention [CDC], 2014b). Multiple drug resistance (MDR) was defined as resistance to three or more classes of antimicrobials. For all tetracycline resistant strains, the presence of *tet(O)* that confers resistance to tetracycline in *Campylobacter*, was detected using a published PCR protocol (Gibreel et al., 2004).

### Multilocus Sequence Typing (MLST)

PCR amplification of seven multilocus sequence typing (MLST) genes was performed using the Kapa2G fast PCR kit (KapaBiosystmes, Boston, MA, USA) with primers listed on the *C. jejuni* and *C. coli* PubMLST website^1^. Amplified products were cleaned using the QIAquick PCR purification kit (Qiagen, Valencia, CA, USA) and sequenced at the Research Technology Support Facility at Michigan State University. Sequences were assembled and checked for overall quality using the SeqMan program in the Lasergene software suite (DNASTAR Inc., Madison, WI, USA), while alleles, STs, and clonal complex (CC) assignments were made using the PubMLST database (Jolley and Maiden, 2010). New alleles (*n* = 2) and STs (*n* = 8) identified in this study were deposited in the PubMLST database (ids 32712-32721, 33135-33155, 33157-33260). A Neighbor-joining tree (*p*-distance) with 1,000 bootstrap replications was constructed in MEGA 6 (Tamura et al., 2013) to examine the evolutionary relationships between *C. jejuni* strains. Clusters were classified for STs that grouped together with >75% bootstrap support, and were further evaluated for genetic recombination using Splitstree4 (Huson and Bryant, 2006).

### Repetitive Sequence-Based PCR (Rep-PCR)

To compare the *C. jejuni* recovered from cattle within the same herd and compare a subset of cattle- and human-derived isolates, rep-PCR was utilized with ERIC1R-ERIC2 primers as described (Patchanee et al., 2012). Template DNA concentrations were standardized to 25 ng/μL prior to PCR and 0.8 μM of each primer was used with the KAPA2G Fast Readymix at the following cycling conditions: one initial cycle at 95°C for 5 min, 35 cycles of denaturation at 94°C for 45 s, annealing at 52°C for 1 min, and extension at 65°C for 10 min, with a single final extension cycle at 65°C for 20 min. The amplified products were separated by electrophoresis at 80 V for 2 h using a 1.5% agarose gel. Fingerprint patterns were analyzed visually and the Dice coefficient with a 2.0% band position tolerance was used to calculate the similarity matrices in Bionumerics ver. 5.10 (Applied Maths, Inc., Austin, TX, USA). Dendrograms were created using the unweighted pair group method with arithmetic averages (UPGMA).

### Data Analysis

Statistical analyses were performed using SAS version 9.3 (SAS Institute, Cary, NC, USA). The χ^2^ test was used to examine differences in the prevalence of *C. jejuni* and antimicrobial resistance between farms and identify associations between antimicrobial resistance profiles and molecular traits (e.g., ST, CC, and cluster) as well as farm characteristics. The Fisher’s exact test was used for variables with ≤5 in at least one cell. Odds ratios (OR) and their 95% confidence intervals (CI) were calculated in EpiInfo version 7.2.0.1^2^ and SAS; *P* < 0.05 was considered significant.

## Results

### Prevalence of *Campylobacter* in Cattle from Three Herds

The prevalence of *Campylobacter* was 71.0% among the 214 cows examined at the three farms. Most of the *Campylobacter* isolates were classified as *C. jejuni* (97.4%; *n* = 148), however, one *C. coli* isolate was recovered from Farm A. Four isolates could not be speciated and were therefore classified as neither *C. jejuni* or *C. coli*. Among all three farms, the highest prevalence of *C. jejuni* was observed at Farm C (83.8%), which was significantly higher than both Farm A (58.6%) and Farm B (62.2%) (*P* < 0.01) (**Table 1**). Virtually all of the animals in Farms B (*n* = 82/83) and C (*n* = 74/75) were sampled, while only 10.9% (*n* = 58/530) of the total animals were sampled at Farm A, which had a considerably larger herd size. After culture and speciation, only 135 of the 148 (91.2%) *C. jejun*i strains were viable and could be tested for susceptibility to nine antimicrobial agents.

**Table 1:** Prevalence of *Campylobacter jejuni* in cattle and antimicrobial resistance frequencies by farm.

Farm	Farm type	Total No. of cattle	No. (%) of cattle sampled		No. (%) of cattle with *C. jejuni*		OR	95% CI	*P*	No. (%) of animals with resistant *C. jejuni*^∗^		OR	95% CI	*P*^∗∗^
A	Dairy	530	58	(10.9%)	35	(58.6%)	ref	–	–	10	(40.0%)	ref	–	–
B	Beef	83	82	(98.8%)	50	(61.0%)	1.0	0.52, 2.04	0.94	45	(90.0%)	13.5	3.98, 45.83	<0.00001
C	Beef	75	74	(98.7%)	62	(83.8%)	3.4	1.51, 7.65	0.002	58	(96.8%)	43.5	8.60, 220.01	<0.00001

*OR, odds ratio; ref, reference group; 95% CI, 95% confidence interval; P, P-value. ^∗^Ten of the 35 isolates from Farm A and 2 of the 62 isolates from Farm C were not viable and were excluded from the analyses. ^∗∗^Fisher’s exact test P-value.*

### Antimicrobial Resistance Profiles of *C. jejuni* Isolates across Farms

Among the 25, 50, and 60 *C. jejuni* strains tested from Farms A, B, and C, respectively, 22 (16.3%) were susceptible to all nine antimicrobials (pan-susceptible), while 113 (83.7%) were resistant to one or more agent. Tetracycline resistance predominated (*n* = 113; 83.7%) followed by nalidixic acid (17.0%) and ciprofloxacin (16.3%). Among the tetracycline resistant isolates, 89 (78.8%) were exclusively resistant to tetracycline, while the remainder (*n* = 24; 21.2%) were resistant to one or more additional antimicrobial (**Table 2**). Twenty one of these tetracycline resistant strains were also resistant to ciprofloxacin and nalidixic acid (CipNalTet), while one strain was only resistant to gentamicin in addition to the tetracycline. Resistance to the macrolides, azithromycin, and erythromycin, was observed in two strains (1.5%), which were also resistant to tetracycline and another antimicrobial class; these strains were classified as MDR. All 113 tetracycline resistant isolates harbored *tet(O)*.

**Table 2:** Resistance profiles of *C. jejuni* recovered from cattle by farm.

Antimicrobial class	Resistance profile	Total		Farm A		Farm B		Farm C	
		No.	(%)	No.	(%)	No.	(%)	No.	(%)
0	Susceptible	22	(16.3)	15	(60.0)	5	(10.0)	2	(3.3)
1	Tet	89	(65.9)	4	(16.0)	28	(56.0)	57	(95.0)
2	TetGen	1	(0.7)	0	(0)	1	(2.0)	0	(0)
2	CipNalTet	21	(15.6)	6	(24.0)	15	(30.0)	0	(0)
3	AziEryNalTet	1	(0.7)	0	(0)	0	(0)	1	(1.7)
5	AziEryCipNalTetTelClin	1	(0.7)	0	(0)	1	(2.0)	0	(0)
	Total	135		25		50		60	

*No., Number; Tet, tetracycline; Gen, gentamicin; Cip; ciprofloxacin; Nal, nalidixic acid; Azi, azithromycin; Ery, erythromycin; Tel, telithromycin; Clin, clindamycin.*

Differences in resistance frequencies were observed between the three farms with dairy Farm A having a significantly lower frequency of resistant *C. jejuni* than the two beef farms (*P* < 0.00001). The frequency of resistance to specific antimicrobials and antimicrobial classes also varied by farm. Farm C, for instance, had a significantly higher proportion of strains (95.0%) resistant solely to tetracycline (Tet) when compared to both Farms A (16.0%) and B (56.0%) (Fisher’s exact *P* ≤ 0.00001). The frequency of any Tet resistance, which included an additional 24 strains with varying profiles, did not differ between Farms C (97.7%) and B (90.0%) (Fisher’s exact *P* = 0.24). Farms C and B combined, however, were more likely to have any Tet resistance than the dairy farm (OR: 22.1; 95% CI: 7.29, 66.80) as only 40% of the 25 Farm A isolates were resistant. By contrast, Farm B had the highest rate (32%) of resistance to CipNalTet when compared to Farms A (24%) and C (0%). Although the frequency of CipNalTet was not significantly higher in Farm B relative to Farm A (OR: 1.4; 95% CI: 0.45, 4.07), the beef Farm B had significantly more CipNalTet resistance than the beef Farm C (Fisher’s exact *P* ≤ 0.00001) and compared to Farms A and C combined (OR: 6.2; 95% CI: 2.23, 17.20). Associations could not be examined for macrolide resistance as only two resistant strains were identified.

### Associations between Farm Characteristics and *C. jejuni* Resistance

In the univariate analysis, significant associations were observed between farm characteristics and the presence and type of antimicrobial resistant *C. jejuni* (Supplementary Table S2). Farms classified as beef operations (OR: 22.1, 95% CI: 7.29, 66.80) or that reported use of preventive antimicrobials like chlortetracycline (OR: 10.5, 95% CI: 2.35, 47.24) were significantly more likely to have resistance to any of the antimicrobials evaluated. On the contrary, use of a mixture of antibiotics for treatment (OR: 0.1, 95% CI: 0.02, 0.42), fly control on the farm (OR: 0.1, 95% CI: 0.02, 0.14), and power washing (OR: 0.1, 95% CI: 0.02, 0.42) significantly lowered the likelihood of resistant *C. jejuni*. For CipNalTet resistance, associations were identified with Holstein breed (OR: 5.6, 95% CI: 2.02, 15.76), mixed antibiotic use for treatments (Fisher’s *P* < 0.0001) and power washing (Fisher’s *P* < 0.0001) in the univariate analysis. Because only three farms were sampled and management practices vary depending on the type of cattle operation studied, our ability to conduct a more comprehensive, multivariate analysis was limited in this study.

### Genetic Diversity and Frequency of *C. jejuni* Genotypes in Cattle

**FIGURE 1:**
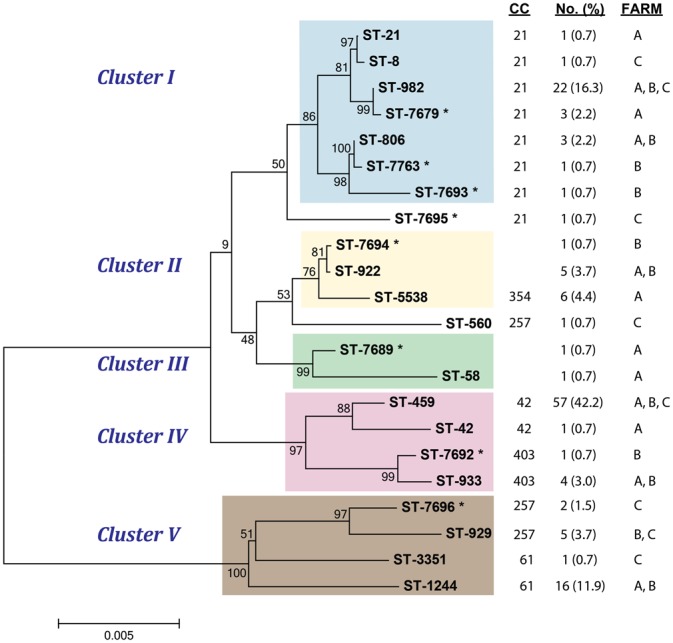
**Neighbor-joining phylogeny of sequence types (STs) recovered from cattle at three farms.** The cluster is noted as well as the previously defined clonal complex (CC) and distribution of strains representing each lineage. ^∗^denotes novel STs recovered in this study.

Multilocus sequence typing was used to investigate the genetic diversity of *C. jejuni* strains recovered from cattle in all three farms. A total of 22 different STs, including eight novel types, were represented among the 135 *C. jejuni* strains recovered. Eighteen of the 22 STs were assigned to six previously defined CCs, while the remaining four STs were classified as singletons. The Neighbor-joining algorithm grouped all 22 STs in to five clusters (I–V) with bootstrap values > 75% (**Figure 1**). The four STs, which were classified as singletons via PubMLST, grouped together into two distinct clusters, Clusters II and III. Cluster III contained one genotype (ST-5538) that was previously classified as CC-354. It is notable that strains representing multiple CCs defined in PubMLST were found to group together within a given cluster. For example, Cluster IV contains strains representing CCs 42 and 403, which grouped together with 97% bootstrap support, while CCs 257 and 61 of Cluster V grouped together with 100% support. All of the STs comprising Cluster I were assigned to CC-21, though one CC-21 isolate was classified as a singleton in this analysis. Although the Neighbor-net analyses on all sites and 162 parsimonious informative sites indicated significant evidence of recombination among the 22 STs [pairwise homoplasy index (PHI) = 0.0], the five clusters identified in the Neighbor-joining phylogeny were similar in the Neighbor-net phylogeny (Supplementary Figure S1).

Among the 135 *C. jejuni* isolates, the most prevalent STs were ST-459 (*n* = 57) and ST-982 (*n* = 22). Both STs were found in all three farms (**Figure 1**) though the frequency varied. For ST-459, Farm C had the highest number of strains (*n* = 35) followed by Farm B (*n* = 20). Farm C also had the highest number of ST-982 strains (*n* = 17), while Farms A and B had 4 and 1 strains, respectively. ST-1244 (*n* = 16) was recovered from Farms A and B, however, a significantly greater number of strains were found at Farm B (*n* = 13). STs 922 (*n* = 5), 933 (*n* = 4), and 806 (*n* = 3) were also observed at Farms A and B, while ST-929 (*n* = 5) was found at Farms B and C. The remaining 15 STs identified were exclusive to specific farms and most were represented by only one strain. Notably, ST-5538 (*n* = 6) was found only at dairy Farm A and two of the novel STs, ST-7679 (*n* = 3) and ST-7696 (*n* = 2), were recovered only from Farms A and C, respectively. Among the previously defined CCs, CC-42 predominated (*n* = 58; 42.9%) in this study followed by CC-21 (*n* = 33; 24.4%) and CC-61(*n* = 17; 12.6%). In all, Farm C had the lowest level of genetic diversity with eight STs, while 12 STs were recovered from Farm A and 11 were recovered from Farm B.

### Association between Phylogenetic Lineage and Antimicrobial Resistance Profiles

**FIGURE 2:**
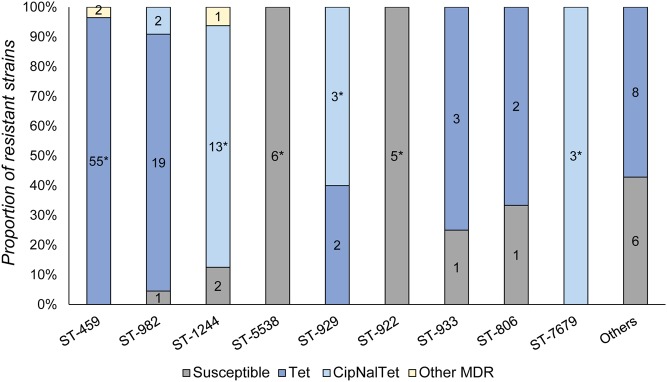
**Distribution of resistance profiles in *Campylobacter jejuni* strains by ST.** Number of isolates belonging to each resistance profile are noted within each bar and ^∗^denotes significant associations between a ST and specific antimicrobial resistance profile. Tet, tetracycline resistance; CipNalTet, ciprofloxacin, nalidixic acid and tetracycline resistant; MDR, multiple drug resistance.

Several significant associations were observed between specific STs and antimicrobial resistance profiles (**Figure 2**). The most prevalent ST detected, ST-459 (*n* = 57), was significantly associated with tetracycline resistance (OR: 22.00; 95% CI: 3.28, 923.78) as the majority (*n* = 55; 96.5%) of strains were resistant to tetracycline only. Most of the 22 isolates assigned to ST-982 (*n* = 21; 95.5%) were also resistant to tetracycline (**Figure 2**), although the association was not significant in the overall analysis because of the high proportion of tetracycline resistance among ST-459 strains. For fluoroquinolone resistance, isolates belonging to ST-1244 were more commonly resistant to ciprofloxacin and nalidixic acid (Fisher’s *P* < 0.0001); 14 of the 16 ST-1244 strains were resistant to both antimicrobials. Thirteen of these strains were also resistant to tetracycline, yielding a significant association between ST-1244 and CipNalTet resistance (Fisher’s *P* < 0.0001). Similarly, strains assigned to ST-929 had a higher likelihood of resistance to CipNalTet (Fisher’s *P* < 0.05) as did isolates assigned to ST-7679 (Fisher’s *P* < 0.01). Significant associations were also observed between specific STs and pan-susceptibility, as all of the strains belonging to ST-5538 (*n* = 6) and ST-922 (*n* = 5) were susceptible to all antimicrobials evaluted (Fisher’s *P* < 0.0001) (**Figure 2**). The associations between ST-459 and Tet resistance as well as ST-1244 and CipNalTet resistance remained significant even when the farm was considered.

Similar associations were observed across the clusters identified in the Neighbor-joining phylogeny. Cluster IV, for instance, mostly consisted of ST-459 (*n* = 57) strains and was significantly associated with tetracycline resistance (OR: 13.1; 95% CI: 2.91, 118.53). Likewise, Cluster V was associated with CipNal (Fisher’s *P* < 0.0001) and CipNalTet resistance (Fisher’s *P* < 0.0001) as ST-1244 (*n* = 16) comprised the majority (66.7%) of strains within this cluster. On the other hand, Cluster II, which is mostly comprised of ST-5538 and ST-922, was significantly associated with pan-susceptibility (Fisher’s *P* < 0.0001) as was Cluster III (Fisher’s *P* < 0.01), though the number of strains in the latter group was small (*n* = 2).

### DNA Fingerprinting Analysis of *C. jejuni* Isolates to Investigate Transmission

**FIGURE 3:**
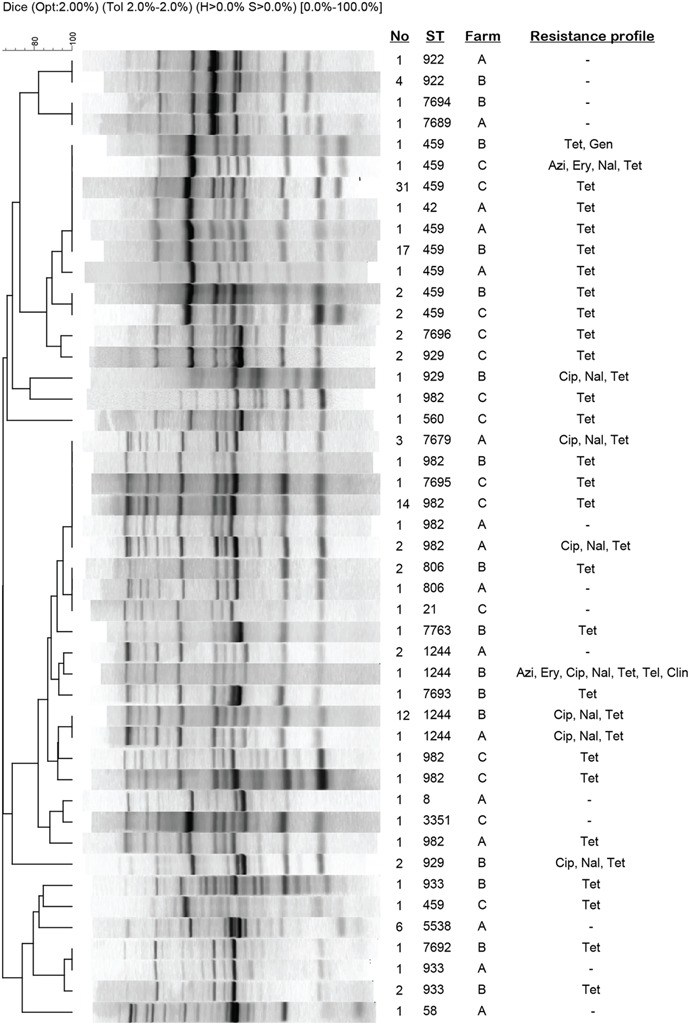
**Repetitive sequence-based PCR profiles of *C. jejuni* strains recovered from cattle.** Patterns represent strains of a given ST that vary by resistance profile or farm. If the same ST was recovered from more than one farm, then both strains were included for comparison. The number (No) of strains representing each pattern and ST is indicated. Azi, azithromycin; Cip, ciprofloxacin; Clin, clindamycin; Ery, erythromycin; Gen, gentamicin; Nal, nalidixic acid; Tel, tetracycline; Tel, telithromycin.

Rep-PCR was performed on all 135 *C. jejuni* isolates to assess the genetic diversity of isolates that were assigned to the same STs, and examine transmission of *C. jejuni* within and between farms. Overall, the fingerprinting patterns correlated well with the MLST results and most strains assigned to the same STs had identical patterns or clustered together (**Figure 3**). Strains belonging to ST-922 (*n* = 5), for instance, were indistinguishable by rep-PCR despite being found at two different farms; the same was true for strains representing ST-806 (*n* = 3), ST-933 (*n* = 2), and ST-1244 (*n* = 16). Most of the STs within each farm were identical by rep-PCR, though there were exceptions as six pairs of strains with different STs had identical fingerprint patterns (e.g., ST-806 and ST-21).

**FIGURE 4:**
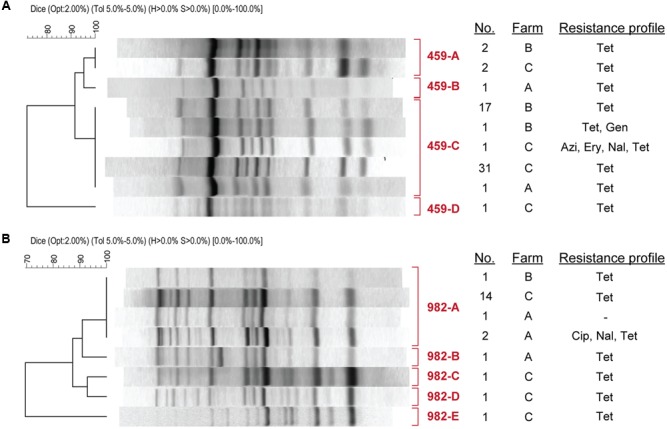
**Repetitive sequence-based PCR pattern of (A)** ST-459 strains and **(B)** ST-982 strains. Patterns represent strains of the same ST that vary by resistance profile or farm. The number (No) of strains representing each pattern (labeled in red) is indicated. Azi, azithromycin; Cip, ciprofloxacin; Ery, erythromycin; Gen, gentamicin; Nal, nalidixic acid; Tel, tetracycline.

The most common genotypes, ST-459 and ST-982, grouped into two major clusters. When each ST was examined by UPGMA analysis separately, most ST-459 strains shared a fingerprinting pattern (459-C) consisting of nine bands on average regardless of the resistance profile and farm of origin (**Figure 4A**). Even though several unique patterns differing by 1–8 bands were observed for these ST-459 strains, only one strain clustered outside of the main ST-459 cluster (**Figure 3**). Importantly, the 459-C pattern representing strains with varying resistance profiles was found at all three of the farms, although the 459-C strains with resistance to only tetracycline varied in prevalence across farms with most coming from Farms B (*n* = 17) and C (*n* = 31).

A similar result was observed for the 22 ST-982 strains with most possessing the same rep-PCR pattern (982-A). Four other patterns represented by one strain each (**Figure 4B**) were clustered together in a different part of the phylogeny. Although strains with the 982-A rep-PCR pattern were found at all three farms, they varied by resistance profile. Farms B and C had 982-A strains with only tetracycline resistance, while strains from Farm A were either pan-suceptible (*n* = 1) or resitant to CipNalTet (*n* = 2). Interestingly, all three CipNalTet resistant isolates belonging to ST-7679 were recovered from Farm A and had the same fingerprint pattern as 982-A (**Figure 3**). This genotype differed from ST-982 by one single nucleotide polymorphism (SNP) in one of the seven MLST housekeeping genes.

### Comparison of *C. jejuni* Isolates from Humans and Cattle and Associations with Antimicrobial Resistance Profiles

A Neighbor-joining phylogeny was also constructed to compare the evolutionary relationships of 94 *C. jejuni* strains recovered from human patients to the 135 cattle strains. The genetic diversity was lower in the cattle strains as 22 cattle-derived STs were identified relative to to the 49 human-derived STs (Supplementary Figure S2), though eight STs (ST-8, ST-21, ST-982, ST-806, ST-922, ST-459, ST-42, and ST-929) were found in both the humans and cattle. Although the bootstrap support was not high enough to identify specific clusters in the combined analysis, the more closely related STs were assigned to the same CCs by PubMLST. Notably, four of the eight overlapping STs (ST-21, ST-8, ST-982, and ST-806), were assigned to CC-21.

**FIGURE 5:**
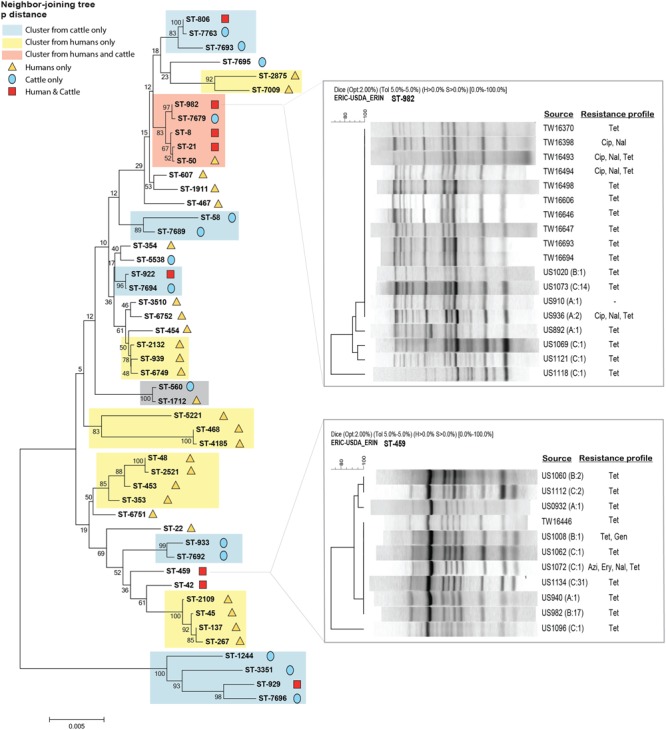
**Neighbor-joining phylogeny of STs recovered among cattle and human patients from Michigan.** Clusters are colored based on bootstrap support for STs within each cluster and source as some clusters are specific only to humans (yellow) or cattle (blue) and others overlap (orange). The gray box highlights a new cluster containing strains from both sources. Rep-PCR banding patterns are shown for the predominant overlapping STs, ST-982 (*n* = 10) and ST-459 (*n* = 1), representing strains from both humans and cattle. Identification numbers starting with TW were recovered from humans, while numbers starting with U.S. are from cattle. The farm and number of cattle strains (No) representing each pattern are indicated in parentheses.

Another phylogenetic tree was constructed to compare the 22 cattle-derived STs to the 36 STs representing strains recovered only from patients residing in Michigan; STs from patients reporting travel outside of Michigan were excluded. The Neighbor-joining phylogeny demonstrated adequate bootstrap support (>78%) for 12 clusters (**Figure 5**). The eight STs that were shared between both sources were included in the analysis, and three of these STs (ST-8, ST-21, and ST-982) were assigned to CC-21 by PubMLST. STs 806, 922, and 929 grouped together in to cattle-specific clusters, while STs 459 and 42 did not cluster together with bootstrap support exceeding 78%. Rep-PCR of human ST-982 (*n* = 10) and ST-459 (*n* = 1) strains were similar to the cattle-derived strains representing the same ST (**Figure 5**, inset). The human ST-982 and ST-459 strains had fingerprint patterns 982-A and 459-C, respectively, which predominated among the cattle-derived isolates recovered from each of the three farms.

The antimicrobial resistance profiles of the eight STs shared between humans and cattle were also examined (Supplementary Figure S3). Notably, similar resistance profiles were observed among strains with the same STs. All strains belonging to ST-21, for example, were pan-susceptible, while ST-42 and ST-459 strains were mostly resistant to tetracycline. ST-982, which consisted of 10 strains from humans and 22 from cattle, also shared a similar resistance pattern. In detail, the majority of strains (81.3%) representing ST-982 were resistant to tetracycline only, however, two ST-982 isolates from both species were CipNalTet resistant. Combining all of the susceptibility and genotyping data from cattle (*n* = 135) and humans (*n* = 94) confirmed the associations identified separately for each species. Specifically, ST-982 was significantly associated with tetracycline resistance (OR:6.41; 95% CI: 1.53, 56.83), while STs 21, 922, and 5538 were more likely to be pan-susceptible (Fisher’s *P* < 0.01). ST-464 and ST-1244, which were found exclusively in humans and cattle, respectively, were significantly associated with CipNal and CipNalTet resistance (Fisher’s *P* < 0.01). The same was true for ST-459 and resistance to tetracycline (OR:14.75; 95% CI: 3.64, 128.14) and ST-7679 with CipNalTet resistance (Fisher’s *P* < 0.01).

## Discussion

This study demonstrates a high prevalence (69.2%) of *C. jejuni* in cattle, indicating that cattle are an important reservoir in Michigan. Because seasonality has been shown to affect *C. jejuni* prevalence in cattle with higher peaks in warmer months (Grove-White et al., 2010), it is possible that higher temperatures contributed to the high frequencies observed given that sampling took place during the summer. Among the three farms sampled, Farm C, a beef operation, had a significantly higher proportion of cattle with *C. jejuni* compared to Farm A (dairy) and Farm B (beef). Previous studies have reported a higher prevalence of *C. jejuni* in beef compared to dairy cattle (Englen et al., 2007) as well as an increased frequency in feedlot cattle over time during their feeding period (Minihan et al., 2004; Besser et al., 2005). It is therefore possible that the significant difference observed between Farm C and other farms in this study is due to the different operation type, proportion of the herd sampled (Farm A) or sampling times (Farm B). Longitudinal studies in additional cattle farms, which utilize a standardized sampling scheme, are warranted to better quantify the prevalence of *C. jejuni* by operation over time.

A high frequency of genotypes ST-459, ST-982, and ST-1244 was observed among the cattle-derived *C. jejuni* isolates. These STs are classified as CC-42, CC-21, and CC-61, respectively, which are the most commonly reported lineages from cattle around the world (Minihan et al., 2004; Kwan et al., 2008; de Haan et al., 2010). According to the PubMLST database, more than 60% of the reported *C. jejuni* isolates from cattle can be classified into these three CCs, suggesting that the genotypes may be better adapted to cattle and the farm environment. To enhance our ability to assess the genetic diversity of *C. jejuni* and investigate transmission of strains with the same ST within and across farms, we utilized rep-PCR, a highly discriminatory tool for characterizing *Campylobacter* (Wilson et al., 2009; Patchanee et al., 2012). Although another single primer targeting (GTG)_5_ was used to increase the discriminatory power, ERIC1R-ERIC2 primers yielded higher discriminatory power for all STs examined in this study. The rep-PCR patterns by ERIC primers correlated well with the STs, confirming that this genotyping tool is useful for epidemiological studies, particularly those conducted in resource limited settings. It is important to note, however, that some discrepancies were found as isolates with different STs were indistinguishable by rep-PCR. Nevertheless, these STs were closely related in the phylogenetic analysis and belonged to the same CCs. Rep-PCR was particularly useful in differentiating the isolates assigned to the predominant STs, ST-459 and ST-982, and showed that some fingerprint patterns were shared across all three farms. One similarity among the farms was the reported contact with other animals including dogs, cats, and wildlife like starlings, pigeons, raccoons, deer (Supplementary Table S1), which could be important for pathogen transmission. Because a recent study in Ohio detected the same genotypes circulating in cattle and starlings in the same area (Sanad et al., 2013), it is possible that some strains are more readily transmitted between farms via birds or other wildlife. Besides ST-459 and ST-982, however, the other STs were detected in Farms A and B or B and C. Since Farms A and C were 62 miles away from north to south and Farm B was located roughly in between (24 miles south of A and 37 miles north of C), more frequent transmission may have occurred between farms in close proximity.

The significantly higher frequency (95%) of tetracycline resistant isolates recovered from Farm C is notable and may be partly due to the distribution of circulating genotypes. Tet resistance was associated with ST-459, or ST-459-C by rep-PCR, and was found at all three farms, though the Farm C beef operation had the highest prevalence. Importantly, Farm C was the only farm to report the use of chlortetracycline, which was added to the feed of new animals upon arrival and was continuously administered at 2 grams/head for 5 days per month. It is therefore possible that the selection pressure associated with chlortetracycline use at Farm C resulted in the dissemination of one Tet resistant genotype that is well adapted to cattle and the environment. This genotype may also be responsible for the high prevalence of *C. jejuni* at Farm C and is in line with previous studies that have reported high levels of tetracycline resistance in *C. jejuni* from cattle given therapeutic and subtherapeutic doses of the antimicrobial (Inglis et al., 2005; Asai et al., 2007). Farm C also reported use of disinfectant spraying as the sole cleaning method every 6 months, which differed from the other farms that used power washing more frequently (Supplementary Table S1). Indeed, a previous study in cattle farms has reported more frequent cleaning of water troughs in barns reduced the risk of *Campylobacter* (Ellis-Iversen et al., 2009). Additional molecular epidemiological studies are greatly needed to further investigate the association between farm management practices in cattle operations, especially antibiotic use and cleaning protocols, and the frequency of *C. jejuni* and antimicrobial resistant *C. jejuni*.

The frequency of macrolide resistance (2%) observed in this study was similar to prior studies in the U.S. with ranges between 0 and 2.9% (Sato et al., 2004; Bae et al., 2005). A higher frequency (16%) of fluoroquinolone resistance, however, was observed relative to other cattle studies (range: 0.6 and 5.0%). Because none of the farms reported use of fluoroquinolones, these data suggest that resistance is maintained in the population in the absence of antimicrobial use. This hypothesis is supported by studies involving the mechanism of fluroquinolone resistance, which typically involves a single point mutation in the chromosomal *gyrA*. Indeed, fluoroquinolone resistant *C. jejuni* strains were found to have increased fitness in chickens even when the selective pressure was removed (Luo et al., 2005). In support of this finding, Farm B had the highest frequency of fluoroquinolone resistance (69.6%), which was associated with one genotype (ST-1244). The clonal spread of fluoroquinolone resistant ST-1244 in Farm B warrants further investigation to explore the *in vivo* fitness of fluoroquinolone resistant *C. jejuni* in cattle and the continuous monitoring of resistance frequencies in the farm environment.

Our previous study with human *C. jejuni* isolates in Michigan showed a significantly higher rate of tetracycline resistance (61.7%) compared to national reports (47.8%) (χ^2^ *P* < 0.01) and further, that resistance was associated with ST-982 and history of cattle contact (Cha et al., 2016b). To our knowledge, this is the first report of a ST-982 *C. jejuni* infection in humans in the U.S., as the ST-982 isolates highlighted in PubMLST were recovered from cattle, a lamb, and the farm environment. Compared to the human strains, which were collected during an overlapping time frame and in the same vicinity as the cattle strains, a significantly higher frequency of tetracycline resistance was observed in the cows (χ^2^ *P* < 0.001). Twenty two cattle strains (16.3%) belonged to ST-982, confirming a high prevalence of this genotype in Michigan cattle. Nineteen (86.4%) of the ST-982 isolates from cattle were resistant to tetracycline, and the association between ST-982 and Tet resistance was significant after combining the human (*n* = 94) and cattle (*n* = 135) isolates (OR: 6.41; 95% CI: 1.53, 56.83). Notably, all 10 ST-982 isolates from humans had the same rep-PCR pattern as the predominant 982-A pattern found in cattle at all three farms. It is important to note, however, that genotypic variation may exist by year and the lack of cattle sampling in 2011 may have limited our ability to detect additional overlapping STs that were present in both species. Nonetheless, these findings suggest that a tetracycline-resistant ST-982 lineage, which has contributed to a high frequency of human infections, is also circulating in Michigan cattle.

Among the CipNalTet resistant isolates from Farm A, we identified a novel genotype, ST-7679, which contains one SNP in one of the seven MLST loci relative to ST-982. Because the rep-PCR patterns for these ST-7679 strains were identical to the ST-982 strain patterns, it is likely that diversification of this resistant lineage has occurred in this farm environment. Further characterization by whole genome sequencing will be useful to elucidate the evolutionary relationship between these two genotypes as well as more comprehensively monitoring transmission patterns within and across herds and humans. Although these isolates were confined to Farm A, ST-982 was widespread in both humans and cattle in Michigan and hence, additional monitoring of these resistant lineages is warranted.

Since this study is cross-sectional in design with samples collected from only three farms, it is possible that our results are not generalizable to all Michigan cattle or even to cattle outside of Michigan. Nonetheless, common associations were identified between resistance profiles and specific lineages in strains from all three farms as well as in humans with campylobacteriosis. Taken together, these data suggest that a subset of resistant genotypes are circulating in both the cattle and human populations in a given geographic location and that cattle serve as an important reservoir for both *C. jejuni* and resistant *C. jejuni*. Given the impact of antimicrobial resistance on human and animal health, it is clear that longitudinal studies are needed to better define risk factors for emergence, persistence, and transmission of resistant *C. jejuni* in cattle and the environment while focusing on those lineages that are more readily linked to human infections.

## Author Contributions

WC, RM, DG, and SM conceived the study and contributed materials; RM, CV-V, SR, PB, DG, and SM planned the study and sampled the animals; WC, RM, and SW performed the experiments; WC and SM analyzed the data and drafted the paper; all authors approved the final version.

## Conflict of Interest Statement

The authors declare that the research was conducted in the absence of any commercial or financial relationships that could be construed as a potential conflict of interest.

## Footnotes

1 http://pubmlst.org/campylobacter

2 www.cdc.gov
