# Supplementary material for: Comparing the Genetic Diversity and Antimicrobial Resistance Profiles of Campylobacter jejuni Recovered from Cattle and Humans
Source: Front Microbiol. 2017 May 9;8:818. doi: 10.3389/fmicb.2017.00818 (PMC5422560; doi:10.3389/fmicb.2017.00818)
Supplement: Supplementary file 1 [file Table_1.DOCX]

**Table S1. Farm characteristics and management practices**

|  |  | | **Farm A** | **Farm B** | **Farm C** |
| --- | --- | --- | --- | --- | --- |
| **Demographics** | **Operation type** | | Dairy | Beef | Beef |
|  | **Number of animals** | | 530 | 83 | 75 |
|  | **Breed** | | Crossbred | Holstein | Crossbred |
|  | **County** | | Clinton | Ingham | Calhoun |
| **Infectious disease preventive measures** | **Antibiotic use in feed or water** | | No | No | Yes  (Chlortetracycline) |
|  | **Any direct fed microbials** | | No | No | Yes  (Yeast mineral package) |
|  | **Antiparasitic** | | Yes (Moxidectin) | Yes (Doramectin) | Yes (Doramectin) |
|  | **Rumensin in the feed** | | No | Yes | Yes |
| **Infectious disease treatments*** | **Respiratory Disease** | | Ceftiofur, Florfenicol | Ceftiofur, Florfenicol Gamithromycin, Tulathromycin | Tulathromycin |
|  | **Foot infection** | | Copper Sulfate | Oxytetracycline | Tulathromycin |
|  | **Arthritis** | | N/A | Oxytetracycline | Tulathromycin |
|  | **Clinical mastitis/ metritis** | | Oxytetracycline, Ampicillin | N/A | N/A |
| **Contact with other species** | **Fly control** | | Yes  (Premise spray) | No | No |
|  | **Dogs** | | Yes | No | Yes |
|  | **Cats** | | No | Yes | Yes |
|  | **Birds** | | Yes  (Starlings, Pigeons) | Yes  (Sparrows, Starlings) | Yes  (Sparrows, Starlings, pigeons) |
|  | **Other animals** | | Yes  (raccoons, rodents, deer) | Yes  (raccoons, rodents, skunks) | Yes  (raccoons, rodents, skunks, opossum, weasel) |
| **Cleaning** | **Method** | | Scrape; Wash/Power Wash; Spread lime | Wash/Power Wash | Spray a disinfectant |
|  | **Frequency** | **Feedbunks** | Once a week | When needed | Once per 6 months |
|  |  | **Waterers** | Once a week | 20 per month | Once per 6 months |
| **Environment** | **Temperature**** | | 85 ⁰F (73 – 97 ⁰F) | 68 ⁰F (62 - 73 ⁰F) | 75 ⁰F (65 – 84 ⁰F) |

* Common remedies used.

** Average temperature on the day of sampling with the minimum and maximum temperature observed on the date.
