# Supplementary material for: Comparing the Genetic Diversity and Antimicrobial Resistance Profiles of Campylobacter jejuni Recovered from Cattle and Humans
Source: Front Microbiol. 2017 May 9;8:818. doi: 10.3389/fmicb.2017.00818 (PMC5422560; doi:10.3389/fmicb.2017.00818)
Supplement: Supplementary file 2 [file Table_2.DOCX]

**Table S2. Univariate analysis of factors associated with any antimicrobial resistant (AR), and Ciprofloxacin, Nalidixic acid, Tetracycline (CipNalTet) resistant *C. jejuni* in cattle belonging to three herds in Michigan**

|  | AR *C. jejuni* with characteristic | | | CipNalTet resistant  *C. jejuni* with characteristic | | | | |
| --- | --- | --- | --- | --- | --- | --- | --- | --- |
| Characteristic | **No. (%)** | **OR (95% CI)** | ***P*** | **No. (%)** | | **OR (95% CI)** | | ***P*** |
| Operation type  Beef  Dairy  Breed  Holstein  Crossbred  Antibiotic use in feed or water  Yes  No  Infectious disease treatment  Mixed antibiotics  One antibiotic  Fly control  Yes  No  Cleaning method  Wash/Power wash  Spray a disinfectant | 103/110 (91.2)  10/ 25 (40.0)  45/50 (90.0)  68/85 (80.0)  58/60 (96.7)  55/75 (73.3)  55/75 (73.3)  58/60 (96.7)  10/25 (40.0)  103/110 (91.2)  55/75 (73.3)  58/60 (96.7) | 22.1 (7.29, 66.80)  Ref  2.25 (0.77, 6.53)  Ref  10.5 (2.35, 47.24)  Ref  0.1 (0.02, 0.42)  Ref  0.04 (0.02, 0.14)  Ref  0.1 (0.02, 0.42)  Ref | <0.0001  0.1287  0.0003  0.0003  <0.0001  0.0003 | 15/110 (13.6)  6/25 (24.0)  15/50 (30.0)  6/85 (7.1)  0/60 (0.0)  21/75 (28.0)  21/75 (28.0)  0/60 (0.0)  6/25 (24.0)  15/110 (13.6)  21/75 (28.0)  0/60 (0.0) | 2.0 (0.69, 5.81)  Ref  5.6 (2.02, 15.76)  Ref  0.0  Ref  Infinity  Ref  2.0 (0.69, 5.81)  Ref  Infinity  Ref | | 0.1969  0.0004  <0.0001  <0.0001  0.1969  <0.0001 | |
|  |  |  |  |  |  | |  | |

No. = number; OR = odds ratio; ref = reference group; 95% CI = 95% confidence interval; *P* = *P*-value
