# Supplementary figures and images for: Comparing the Genetic Diversity and Antimicrobial Resistance Profiles of Campylobacter jejuni Recovered from Cattle and Humans
Source: Front Microbiol. 2017 May 9;8:818. doi: 10.3389/fmicb.2017.00818 (PMC5422560; doi:10.3389/fmicb.2017.00818)

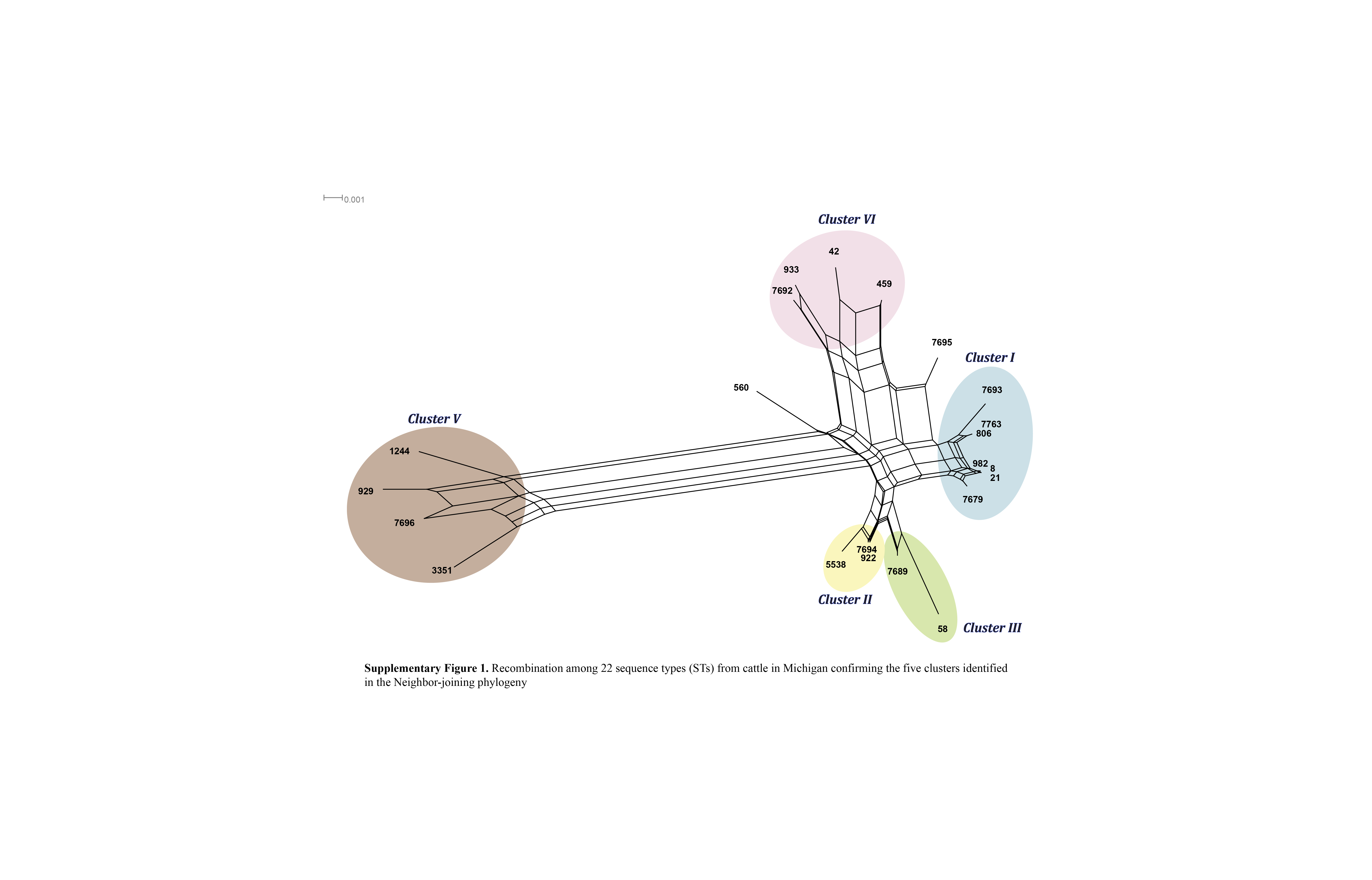

Supplement: Supplementary file 3 [file Image_1.TIF]

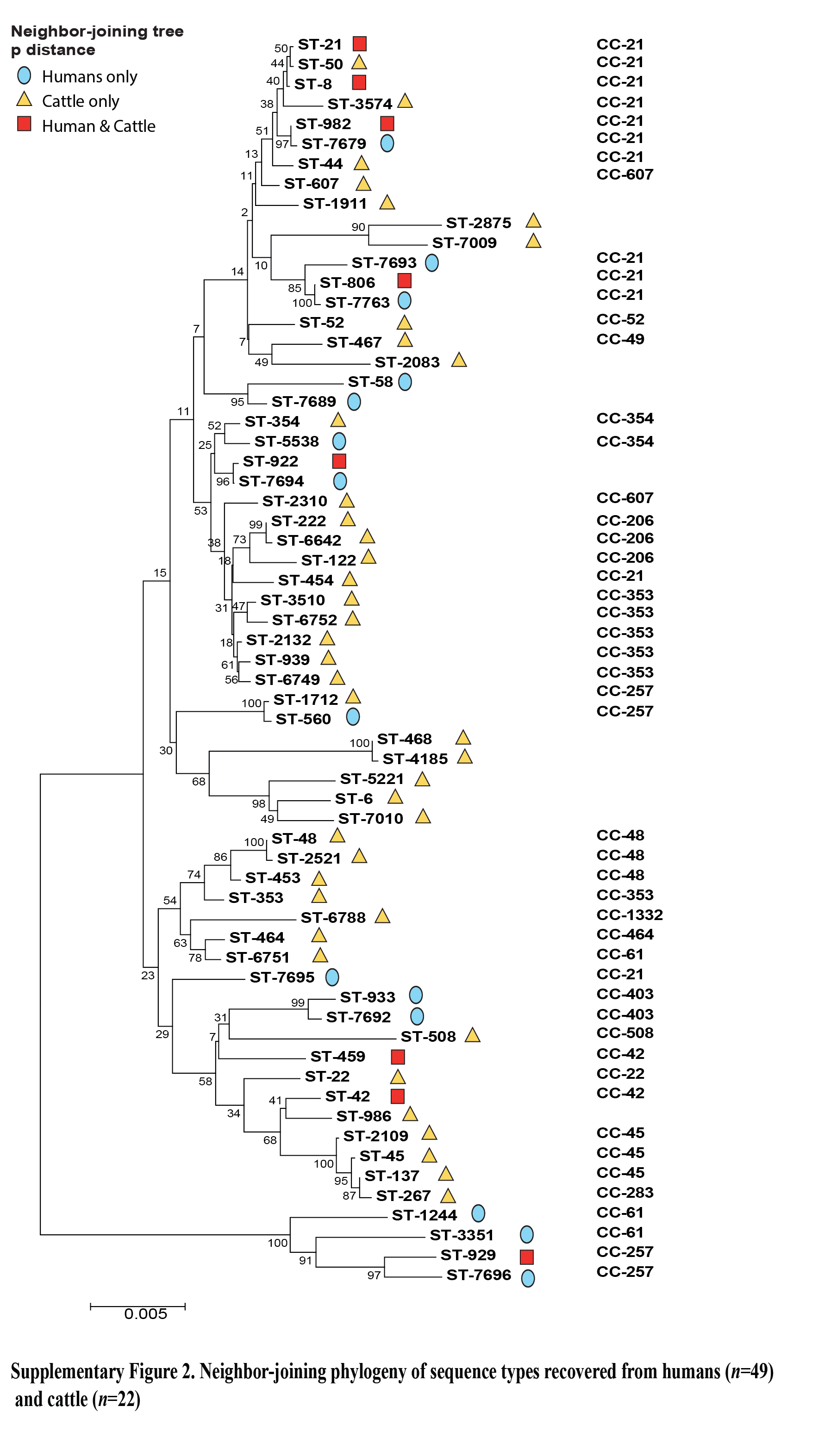

Supplement: Supplementary file 4 [file Image_2.TIF]
