# Supplementary material for: Comparing the Genetic Diversity and Antimicrobial Resistance Profiles of Campylobacter jejuni Recovered from Cattle and Humans
Source: Front Microbiol. 2017 May 9;8:818. doi: 10.3389/fmicb.2017.00818 (PMC5422560; doi:10.3389/fmicb.2017.00818)
Supplement: Supplementary file 5 [file Image_3.PDF]

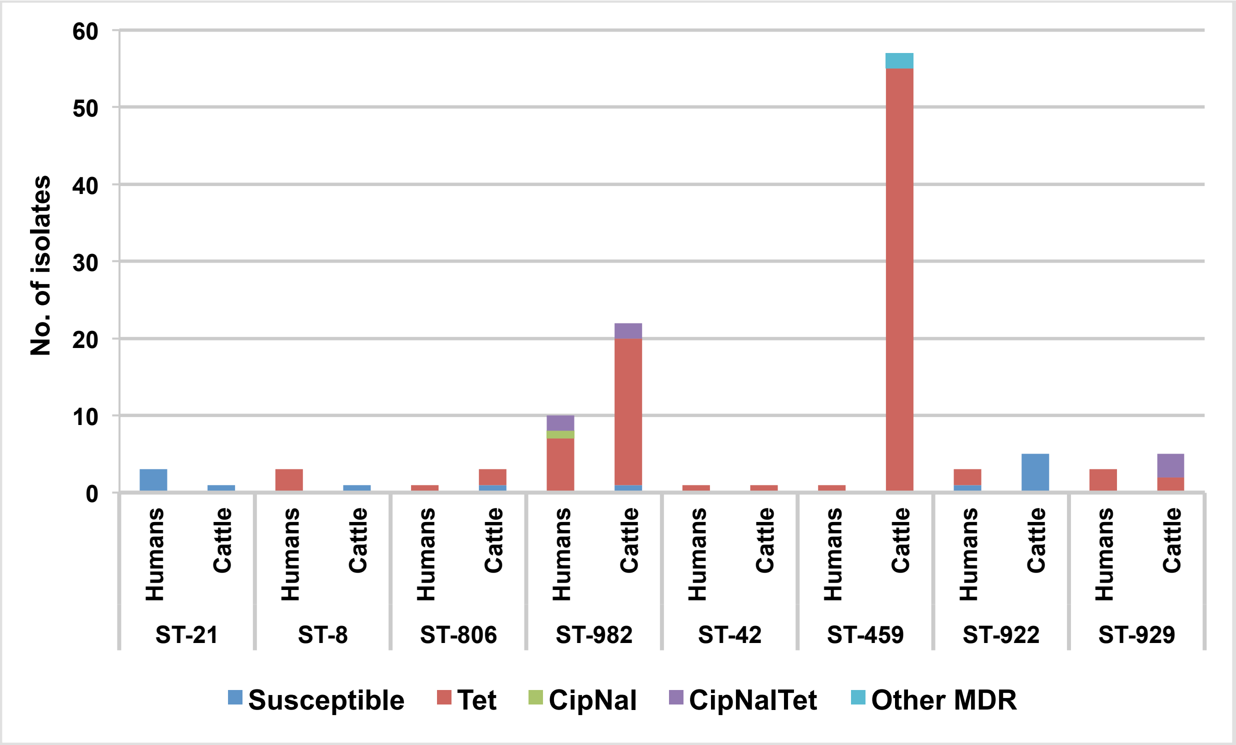

Supplementary Figure 3. Histogram of the antimicrobial resistance profile of STs found both in humans and cattle

Tet = tetracycline resistance, CipNalTet = ciprofloxacin, nalidixic acid and tetracycline resistant; MDR = multiple drug resistance
